# MMR: a tool for read multi-mapper resolution

**DOI:** 10.1093/bioinformatics/btv624

**Published:** 2015-10-30

**Authors:** André Kahles, Jonas Behr, Gunnar Rätsch

**Affiliations:** Memorial Sloan Kettering Cancer Center, Computational Biology Center, 1275 York Avenue, New York, NY 10065, USA

## Abstract

**Motivation:** Mapping high-throughput sequencing data to a reference genome is an essential step for most analysis pipelines aiming at the computational analysis of genome and transcriptome sequencing data. Breaking ties between equally well mapping locations poses a severe problem not only during the alignment phase but also has significant impact on the results of downstream analyses. We present the multi-mapper resolution (*MMR*) tool that infers optimal mapping locations from the coverage density of other mapped reads.

**Results:** Filtering alignments with *MMR* can significantly improve the performance of downstream analyses like transcript quantitation and differential testing. We illustrate that the accuracy (Spearman correlation) of transcript quantification increases by 15% when using reads of length 51. In addition, *MMR* decreases the alignment file sizes by more than 50%, and this leads to a reduced running time of the quantification tool. Our efficient implementation of the *MMR* algorithm is easily applicable as a post-processing step to existing alignment files in BAM format. Its complexity scales linearly with the number of alignments and requires no further inputs.

**Availability and implementation:** Open source code and documentation are available for download at http://github.com/ratschlab/mmr. Comprehensive testing results and further information can be found at http://bioweb.me/mmr.

**Contact:** andre.kahles@ratschlab.org or gunnar.ratsch@ratschlab.org

**Supplementary information:** Supplementary data are available at *Bioinformatics* online.

## 1 Introduction

Addressing the increasing need for fast and accurate mapping of high-throughput sequencing data to a reference sequence, many different software tools have been developed over the past years, many of which are frequently updated and improved (Dobin *etal.*, 2013; Jean *et al.*, 2010; Kim *etal.*, 2013; Li and Durbin, 2009). While numerous challenges have been addressed by the developers, e.g. the consideration of gaps and mismatches or the spliced alignment of RNA-Sequencing data, the problem of ambiguous read mapping still remains unresolved for many of the most popular alignment tools. Depending on factors like read length, alignment sensitivity and repetitiveness of the target genome, a large fraction of reads aligns uniquely to the target and exactly one mapping location is reported. However, for the remaining, still significantly large, fraction of reads (≈10–20%, depending on alignment sensitivity), several possible mapping locations exist. Currently, different strategies are employed to deal with these reads in downstream analyses, most of which have unfavorable side effects: Discarding reads with ambiguous alignments from the alignment result leads to a systematic underestimation of abundance in genomic regions with multi-mapper ambiguities, whereas picking a random alignment or distributing weight across all alignments uniformly does not have a proper biological justification. We provide a brief review of related approaches (Hashimoto *etal.*, 2009; Li *etal.*, 2010; Mortazavi *et al.*, 2008; Wang *et al.*, 2010; Zhang *et al.*, 2013) in Supplementary Section A.5.

Here, we present a simple, yet powerful tool, called the multi-mapper resolution (*MMR*) tool, that assigns each read to a unique mapping location in a way that the overall read coverage across the genome is as uniform as possible. *MMR* makes use of the critical fraction of unambiguously aligned reads and iteratively selects the alignments of ambiguously mapping reads in a way the overall coverage becomes more uniform. MMR was motivated by and developed for post-processing of RNA-Seq alignments in order to improve transcript quantification and prediction. We show that it is also applicable to post-processing DNA-Seq alignments.

## 2 Approach

### 2.1 Outline of algorithm

Our approach to resolve ambiguous mapping locations is based on the simple assumption that, besides all existing biases from library preparation and sequencing that cause coverage differences over a longer range, the alignment coverage should generally be uniform within a local region (RNA-seq or whole-exome-seq) or the whole genome (WGS-seq). On the basis of this assumption, we can evaluate the fit of an alignment of a read to its current mapping location relative to other locations, by assessing the local coverage of the candidate regions. For each read, the algorithm jointly evaluates all available alignments with the goal of selecting the alignment/mapping that results in the smoothest overall coverage. At the beginning, for each read, one alignment is selected based on best alignment score, the given input order or random choice. The set of all initially picked alignments as well as alignments of uniquely mapped reads define a global coverage map. On the basis of this map, we can evaluate the quality of an alignment in its coverage context. To choose the locally optimal alignment for each read, we perform a comparison of all alignments *a* with respect to a loss function $\ell^{+}(a)$ of placing *a* relative to not placing it ($\ell^{-}(a)$). In the simplest case, the loss function is defined as the empirical variance of the read coverage within a window around the alignment (see Supplementary Material). This quantity can be computed efficiently since we keep track of the global coverage map, which is updated when the selected alignment changes. Given the currently selected alignment *a* and an alternative alignment *b*, we update our choice, if the overall loss across the genome would be reduced by choosing the alternative alignment. This is the case when $\ell^{-}(a)+\ell^{+}(b)<\ell^{+}(a)+\ell^{-}(b)$. This is repeated for all reads with ambiguous mapping locations. Several iterations over the whole alignment file improve the results. However, the most striking improvements are achieved within the first three iterations and only slight changes can be observed after that (cf. Supplementary Fig. S9). A more detailed description is provided in Supplementary Section A.

### 2.2 Paired-end reads

Handling paired-end reads in our framework is straightforward: Instead of evaluating two individual mapping locations, the same principle is used to compare two pairs of alignments. After generating a list of proper pairs, where feasibility is determined through orientation, chromosome and reasonable proximity, the list is evaluated the same way as the list of possible mapping locations for single-end reads. This approach is easily adaptable to *n*-tuple of mates for *n* > 2.

### 2.3 Adaptations for RNA-seq data

When mRNA is sequenced instead of DNA, the alignments to the genome show additional complexity caused by the exon/intron structure. In this case *MMR* can incorporate annotation information to compute the loss in local windows that do not overlap the genomic breakpoints implied by the annotated exons. For more details on this, we refer to Supplementary Section A.4.1.

### 2.4 Limiting ambiguity

To find a good trade-off between mapping sensitivity and the number of possible mapping locations, we allow to restrict the list of possible mapping locations. This is achieved by thresholding the difference in edit operations between the best hit and any other alignment. For instance, a filter of 0 would only include alignments as possible mapping locations that have as few edit operations as the best mapping.

### 2.5 Implementation

The *MMR* approach is implemented in C++ and its source code is publicly available at http://github.com/ratschlab/mmr. Although it has been tested and optimized for Linux-based systems, it can be compiled on other platforms. Parsing of alignment files in BAM format requires *samtools* (Li *etal.*, 2009). We also provide a multi-threaded implementation that keeps the coverage information in common memory, requiring no additional memory if multiple threads are used. The single-threaded running time depends on the number of possible mapping locations per read but is on average 30–45 s per one million alignments per iteration. Thus, running *MMR* for three iterations on 100 million alignments takes ≈20 min using 10 threads (Intel Xeon E5-2665 CPU).

## 3 Application

As a proof of principle, we tiled the *A.thaliana* genome with overlapping 50-mers and aligned these 50 nt reads back to the genome. This resulted in a non-uniform coverage, in particular near repetitive regions (see Supplementary Fig. S2). Using *MMR*, we could fully resolve mapping ambiguities, resulting in the expected uniform coverage of 50 almost everywhere.

**Fig. 1.**
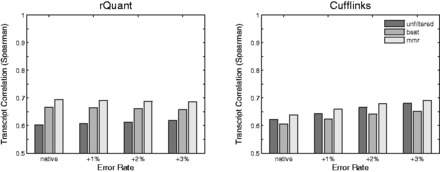
*MMR* results on simulated read data: quantification results on 7 million simulated 51 nt reads aligned with *TopHat2*, using *rQuant* and *Cufflinks.* Accuracy was measured as Spearman correlation to ground truth. Unfiltered read sets are shown in dark, best-hit read sets in medium and *MMR*-filtered in light gray. Native error rate is 0.9%, noise levels 1–3%

Although *MMR* has been successfully used in several studies (Drechsel *et al.*, 2013; Rühl *et al.*, 2012), we wanted to rigorously test our approach on a set of 7 million artificial RNA-Seq reads that were generated with FluxSimulator (Griebel *etal.*, 2012) based on a subset of 5000 genes randomly drawn from the human Gencode annotation (v19). We simulated read sets of length 32 nt, 51 nt, 76 nt and 101 nt, resulting in average coverages between 9× and 28×. The reads were then mutated using an empirical error model that led to a native error rate of 0.9%. Three levels of random noise (+1%, +2%, +3%) were applied in addition. We aligned the reads with *TopHat2* (v2.0.2; Kim *etal.* 2013) and *PALMapper* (v0.6; Jean *etal.* 2010), allowing for up to 6 edit operations, with no annotation provided. Further information is provided in Supplementary Section B.2. To investigate the effect of *MMR* on downstream analyses, we performed transcript quantification using *Cufflinks* (Trapnell *etal.*, 2010) (v2.1.1) and *rQuant* (Bohnert *etal.*, 2009) on the *MMR*-filtered alignments, the best alignments only (the alignment ranked highest by the aligner) and on completely unfiltered alignments. For *TopHat2* and *PALMapper*,** the quantifications based on the *MMR*-filtered alignments showed a consistently better correlation to the ground truth than both the best-hit and unfiltered alignments sets. The shorter reads of length ≤51 nt (Fig. 1) showed larger improvements compared to unfiltered (*Cufflinks*: 2.7%, *rQuant*: 15.0%) and best-hit set (*Cufflinks*: 5.6%, *rQuant*: 4.0%) than the longer reads of length ≥76 nt, that showed consistent but smaller improvements (Supplementary Figs S5–S7).

## 4 Conclusion

We presented *MMR*, a post-processor for BAM files, resolving ambiguous alignment locations. We showed its easy applicability to the output of different alignment methods and illustrated that *MMR* can greatly improve accuracy of downstream quantification methods. Although the improvements seem moderate on a global scale, the effect on single genes can be much larger. Given its lean implementation and the short running time, *MMR* is very well suited for large-scale genome-, exome- and RNA-sequencing efforts. Its good performance on short reads also suggests an application to ribosome footprinting data (Ingolia *etal.*, 2012). *MMR* may also be useful for post-processing alignments in meta-genome projects for improved selection of taxa.

## Supplementary Material

Supplementary Data
