# Supplementary material for: MMR: a tool for read multi-mapper resolution
Source: Bioinformatics. 2015 Oct 30;32(5):770–2. doi: 10.1093/bioinformatics/btv624 (PMC4795617; doi:10.1093/bioinformatics/btv624)
Supplement: Supplementary Data [file btv624_supplementary_data.zip › supplementary.pdf]

Supplementary Material
MMR: A tool for Read Multi-Mapper Resolution

André Kahles,[1] Jonas Behr,[1,‡] and Gunnar Rätsch[1]

[1] Computational Biology Center, Sloan-Kettering Institute, 1275 York Ave, New York, NY 10065, USA
[‡] Current address: ETH Zürich, D-BSSE, Mattenstrasse 26, CH-4058 Basel, Switzerland

A METHODS

A.1 Description of Algorithm

Following the ideas described in the main manuscript and using the assumption of locally smooth coverage, *MMR* evaluates the whole set of possible alignments for each given read. The goal is to identify the one alignment for each read that results in the locally smoothest coverage. There are multiple ways to express our prior knowledge in what the coverage should look like. We assume that this prior knowledge is encoded in a global loss function ℓ that computes the global amount of non-smoothness for a chosen alignment set. In the simplest case, we measure smoothness as the empirical variance of the position-wise coverage in a window of a given length (see (1) below). Other options are discussed in Suppl. Section A.4. The algorithm then minimizes the loss function over all possible alignment choices and chooses the alignment with the smallest overall loss (i.e., greatest "smoothness"). For this, an iterative procedure is applied. Given an input of k different alignments for a given read, one alignment is designated as the currently selected one. Depending on user preference this is either an arbitrary alignment or the mapping with the highest alignment quality (see user documentation). The currently selected mapping is then compared to each of the remaining mapping possibilities. For a single comparison of two alignments a and b, four loss function values are computed: the loss with alignment a placed vs not placed and alignment b placed vs not placed. Given two possible alignments a and b to the genomic start locations p_a and p_b, respectively, the score ℓ_a^+ computes the local loss around genomic location l_a if a is chosen and ℓ_a^- if it is not chosen (i.e., the read is aligned somewhere else); ℓ_b^+ and ℓ_b^- are defined analogously using the alignment b to position p_b.

In the simplest case the loss function is defined as the empirical variance over the genomic coverage of all window positions

$$\ell_a = \frac{1}{L_a - 1} \sum_{i=0}^{L_a-1} \left(a[i] - \frac{1}{L_a} \sum_{j=0}^{L_a-1} a[j] \right)^2 \quad (1)$$

where L_a is the length of the window around alignment (influenced by option `-w`), $a[i]$ indicates the read coverage at position i relative to the start p_a of the alignment a. If an alignment is present within the window, it influences the coverage and thus the local variance or more generally the loss function. After computing all four values, alignment a is chosen if

$$\ell_a^+ + \ell_b^- < \ell_a^- + \ell_b^+,$$

otherwise alignment b is chosen. Figure S-1 shows a schematic of the *MMR* principle. We discuss other loss functions in Suppl. Section A.4.

A.2 Overlapping Alignment Locations

A major complication arising during the computation of ℓ_a^+, ℓ_a^-, ℓ_b^+ and ℓ_b^- is the special case that occurs when the windows of a and b share common positions. In this situation, two different scenarios can occur:

1. the windows share positions but the alignments do not share positions,
2. the alignments share positions.

As the read is placed at either the one or the other location, in case 1) the computation of ℓ_a^- needs to consider coverage contributed by b as this will be placed instead of a and ℓ_b^- needs to consider coverage contributed by a. Case 2) causes a subset of positions that are shared by a and b to not be altered by the decision. These positions can be masked for analysis and left out in computation, as they contribute to both locations not changing the result.

A.3 Paired-end Reads

The approach described above can be easily extended to also work for paired-end alignments. In this case, a preprocessing step iterates through all possible valid pairs of alignments of the two mates (see option `-p`). An alignment pair is valid, if the corresponding alignments do not overlap in a conflicting manner. For instance, a conflict would occur, if the first read-mate is aligned into the intronic portion of the second read-mate, if both reads are aligned in the same direction, if the reads align to different chromosomes, or if both alignments have a distance outside of a user-defined maximum range (see option `-i`). After this preprocessing-step, each alignment pair is treated as single alignment possibility $\mathbf{a}_k$ and the algorithm above is applied. As the number of possible pairs is quadratic in the number of alignments in the worst case, the number of allowed pairs can be limited by the user (see option `-A`).

A.4 Loss Functions based on Segment Annotations

For RNA-seq data, one limitation of the straight-forward strategy described so far is that known transcripts are not taken into account. Especially the exon–intron boundaries show steep changes in coverage, but also within exons a change in coverage can often be explained by a mixed signal from several transcript isoforms that superimpose each other. If the transcripts are (approximately) known, this effect can be accounted for during the optimization process. To include structural information into *MMR*, we devised a strategy that takes transcript annotations and quantifications produced by another tool into account. This method can be applied in an iterative scheme. It starts with transcript isoform prediction/quantifications on the alignments using the best hit. Ambiguous alignments can then be re-evaluated based on the intermediate transcript structure and the estimated transcript expression. The improved alignments can then be used

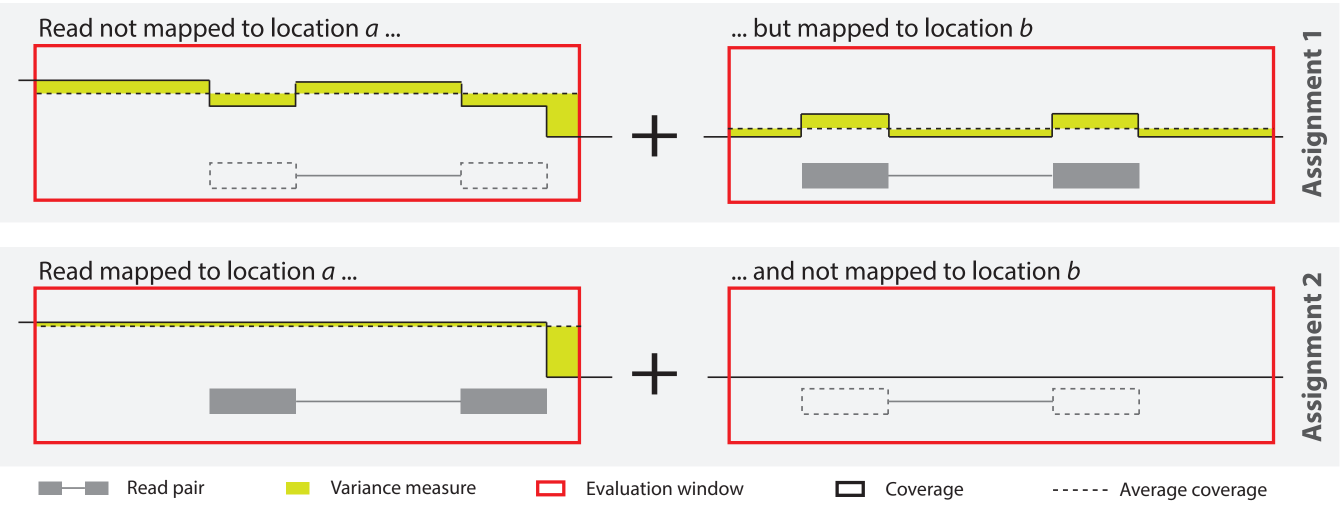

Fig. S-1: **Schematic of the *MMR* Principle —** Schematic overview of the principle to resolve ambiguous read-mappings. The candidate read-pair in gray has two possible alignments a starting at position p_a (left) and b starting at position p_b (right). Loss measures (yellow) are computed for both locations, with and without the read-pair. Loss values from the text have following correspondents in the schema: ℓ_a^- – location a (top), ℓ_a^+ – location a (bottom), ℓ_b^+ – location b (top), ℓ_b^- – location b (bottom). The evaluation windows are shown in red and the coverage of placed reads as black solid lines.

to generate improved isoform predictions and quantifications. This can be repeated a fixed number of times or until convergence of the predicted quantifications. *MMR* allows to input transcript annotations together with transcript quantifications (see option `-s`).

To reposition an ambiguous alignment if transcript structures are given, we devised a strategy for an iterative application of *MMR* and *MiTie* (Behr *et al.*, 2013), a tool for the prediction and quantification of transcript isoforms. If the exon boundaries of all transcript isoforms of a gene are projected to genomic coordinates, the gene can be cut into a set of non-overlapping exonic segments. Thus, each isoform can be built from a subset of these segments. Several isoforms can share the same segment. The expression value of a single segment is the sum of the segment's expression values over all transcript-isoforms containing that segment (for a more formal description we refer to the publication of *MiTie* (Behr *et al.*, 2013)).

The segments as well as corresponding expression estimates provided by *MiTie* or other tools can be used as input for *MMR* (option `-s`). These segments imply a segmentation for the whole genome. Each given segment is associated with a predicted expression value. The empty genomic regions in between any segments are implicitly turned into segments with a predicted coverage of 0. Instead of minimizing the local variance, we now minimize the difference between the observed coverage in an exonic segment with and without the alignment of question and the predicted coverage of the segment.

Again taking the example of the two alignments a and b, for each alignment we can now identify all genomic segments it overlaps with. Let alignment a overlap the m genomic segments $g_{a,1}, \ldots, g_{a,m}$. We can then compute two coverage values for each segment. The value $c_{a,i}^+$ that contains the coverage of segment $g_{a,i}$ if alignment a is mapped to segment i and $c_{a,i}^-$ that contains the coverage of the same segment if a is mapped to a different location. For each segment we can then compute the difference between observed and predicted coverage, using the expression estimates $e_{a,i}$ corresponding to the respective genomic segments $g_{a,i}$ $(i = 1, \ldots, m)$. Thus, the total loss of alignment a is

$$\ell_a^- = \sum_{i=1}^{m} (e_{a,i} - c_{a,i}^-)^2/m \quad \text{and} \quad \ell_a^+ = \sum_{i=1}^{m} (e_{a,i} - c_{a,i}^+)^2/m.$$

Analogously, for alignment b we define the total loss of overlapping genomic segments $g_{b,i}$ with expression estimates $e_{b,i}$ $(i = 1, \ldots, n)$ as

$$\ell_b^- = \sum_{i=1}^{n} (e_{b,i} - c_{b,i}^-)^2/n \quad \text{and} \quad \ell_b^+ = \sum_{i=1}^{n} (e_{b,i} - c_{b,i}^+)^2/n.$$

Besides the different calculation of $\ell_a^+, \ell_a^-, \ell_b^+$ and ℓ_b^-, all other steps are identical to the steps described before. Although slightly different adaptations need to be made in order to account for overlapping alignment locations, the same general principles apply.

A.4.1 Hybrid Strategy We also implemented a hybrid-strategy that does not rely on predicted expression values for the segments and therefore makes *MMR* much more practical. Instead it uses the minimization of the coverage-variance on a segment-basis and computes the sum over all segments an alignment overlaps, to determine a total variance value. For this, the genome is segmented based on exon/intron information taken from a given annotation in GTF/GFF format (see option `-a`). When computing the loss function in local windows, these are now constrained to begin/end at the nearest genomic segment boundary. All other steps are essentially the same as described before.

A.4.2 Other Loss Functions To better account for properties inherent to read-count data, *MMR* can also make use of more general loss functions. For instance, in Behr *et al.* (2013) we used a log-likelihood (loss) function based on a negative binomial distribution. For technical reasons, *MiTie* uses a piece-wise-linear approximation to the log-likelihood loss function. *MMR* supports the specification of an arbitrary piece-wise linear function to score differences between the observed and the expected read coverage (see option `-m`). For further details see user documentation and Behr *et al.* (2013), Suppl. Section K.

A.5 Context of other Methods

As the problem of ambiguous read alignments is not a new one to solve, several other approaches have been followed to make use of this ambiguous data. One of the first tools to assign a unique position to multimappers was part of the *ERANGE* package (Mortazavi *et al.*, 2008), that used a proportional assignment of each read weighted by the evidence of unique alignments at a given locus. Which is a possible but biologically not very well founded design decision. Hence, this strategy has been refined in *MuMRescueLite* (Hashimoto *et al.*, 2009) that completely assigns each multimapper to a single location again based on unique alignments. However, this approach requires a specific input making it difficult to use in standard pipelines and is implemented in Python, which only offers limited room for parallelization. *MMR* accepts standardized BAM input and is implemented in C++, offering multi-threaded computation. Another recent tool, *GeneScissors* (Zhang *et al.*, 2013), uses a machine learning approach to train a random forest classifier that assigns a location to identify expressed fragment attractors. Similar to *MMR* it compares local mapping regions (fragment attractors) and decides where to put a read. However, *GeneScissors* focuses on RNA-Seq data only and needs an additional step of transcript assembly to create candidate fragment attractors. One advantage of this method is, that it also can identify novel alignments that were not suggested by the original aligner. Other approaches, such as *RSEM* (Li *et al.*, 2010), are tied to isoform expression estimation from RNA-Seq data and resolve mapping ambiguity in this context but are not generally applicable to any alignment file in BAM format, or involve a re-mapping of the reads (Wang *et al.*, 2010), further increasing the computational burden.

B RESULTS

As described in the main text, we evaluated *MMR* on two different simulated datasets for an assessment of its properties and performance: a) Whole-Genome Sequencing (WGS) in *A. thaliana* and b) RNA-Seq in human. All results were obtained using the basic version of *MMR* and without any additional inputs, i.e., no annotation or quantification was used for these experiments.

B.1 Post-Processing Alignment of Whole-Genome Sequencing for Improved Repeat Handling

For the first dataset we tiled the complete *A. thaliana* TAIR10 reference genome (Lamesch *et al.*, 2012) at each position into a set of overlapping 50-mers, thus generating artificial reads from whole genome sequencing, including all low-complexity regions of the genome. In this idealized dataset the coverage at each genomic position (except the 50nt at each end) is exactly 50. We then used *PALMapper* to realign the first $1,000,000$ reads back to the *A. thaliana* genome, allowing for up to 5 edit operations, thus generating a high level of additional ambiguity.

As shown in the histograms of coverage distributions in Figure S-2 panel A, *MMR* is able to fully resolve all read-ambiguities in the genomic DNA dataset. In Figure S-2 panel B, we show an example for a genomic region that shows an uneven coverage before *MMR* filtering and is smoothed after filtering. Notably, the unfiltered alignments showed single genome positions with a coverage exceeding $1,700$ (these are contained in the last bin of the histogram in Fig. 1).

As an advantage of the evaluation being based on simulated data, we know for each generated read its genuine position. Thus, we can evaluate if *MMR* helps to pick a correct mapping location for each read. Figure S-3 shows the distribution of alignment ambiguity for each of the aligned reads. While most reads only have few possible mapping locations, several low complexity reads have more than 50 possible mappings. For each read we evaluated two strategies to pick one mapping location as correct: by (a) choosing the best hit alignment determined by the highest alignment score and (b) using the alignment chosen by *MMR*. In Figure S-4 we show the fraction of correct vs. incorrect alignments stratified by alignment multiplicity (the bin for a single alignment location is omitted as no mapping ambiguity arises in this case). The top panel shows the best-hit strategy that mainly picks the wrong alignment and only can pick correctly at all for alignment multiplicities of up to two. The lower panel shows the *MMR* result, which picks the correct mapping location in about 80% of the cases.

B.2 Post-Processing RNA-seq for Accurate Transcript Quantification

The second evaluation dataset, generated using the *FluxSimulator* (Griebel *et al.*, 2012), consisted of sets of 7×10^6 artificial RNA-Seq reads sampled from 5,000 randomly selected genes of the human GENCODE annotation (v19). We simulated a whole set of different read-lengths: 32nt, 51nt, 76nt, and 101nt. Further, we simulated a set of 8×10^6 reads sampled from the full annotation set. All reads were mutated as described below and aligned to the hg19 human reference genome using *TopHat2* (version 2.0.2 (Kim *et al.*, 2013)) and *PALMapper* (version 0.6 (Jean *et al.*, 2010)) allowing up to 6 edit operations without additional annotation information provided. All other parameters were left at their defaults.

Data simulation and error modeling For simulation, we used *FluxSimulator* in its default configuration without using the pre-built error model. As insert size distribution, we provided a normally distributed sample with a mean of 300 and a standard deviation of 100. To best reflect a realistic error pattern, we estimated substitution frequencies from a publicly available RNA-Seq dataset (Lahens *et al.*, 2014). We aligned the data to the human reference genome using *PALMapper*, allowing for 10 edit operations but no spliced alignments. From these alignments, we generated substitution frequencies where we recorded how often a base/quality pair had been substituted by another base. This

results in a characteristic dependency between base calling quality and substitution rate. Using a custom script, the unmutated reads from *FluxSimulator* were than paired with a quality line sampled from the same publicly available RNA-Seq set and substitutions were inserted given the frequency estimated earlier. In addition, we allowed for a 0.1% insertion and deletion probability. In total, this resulted in a native error rate of approx. 0.9%. To model higher noise datasets, we increased the baseline substitution frequency by 1%, 2% and 3%. Table A shows an example of the resulting substitution statistics for a set of 101nt reads from this study.

**Mutation statistics - native error rate**					
	A	C	G	T	N
A	0	567,724	969,811	387,415	11,985
C	242,625	0	314,881	416,398	7,632
G	443,678	332,706	0	227,357	8,708
T	450,255	1,333,391	748,215	0	15,289
insertions					334,733
deletions					210,449
total pos					707,283,608
**Mutation statistics - native error rate + 1%noise**					
	A	C	G	T	N
A	0	1,101,952	1,884,200	752,543	23,143
C	556,735	0	722,969	956,169	17,566
G	1,004,855	751,147	0	515,241	20,040
T	881,458	2,598,584	1,464,425	0	29,887
insertions					685,928
deletions					424,477
total pos					707,283,608

Table A: Substitution statistics for read mutation according to estimated error model for a sample of 7×10^6 reads of length 101. Statistics are shown for two different error rates, the native error rate of approx. 1% and native error rate plus an additional 1% random noise.

Evaluation of simulated data Based on this dataset, we tested the effect of *MMR* on downstream analyses. For this, we used the unprocessed, the *MMR*-filtered and the best-hit alignment set to perform *in silico* transcript quantification using both *cufflinks* (Trapnell *et al.*, 2010) (version 1.3) and *rQuant* (Bohnert *et al.*, 2009), where the best-hit set consisted of those alignments that were ranked highest by the alignment algorithm. Figures S-5 and S-6 show quantification results for a read lengths of 51nt, 76nt and 101nt for different read error levels, and all combinations of the aligners and the quantification tools used. In Figure S-7 we show on overview of quantification results on the read data with native error rate for a set of increasing read lengths ranging from 32nt to 151nt.

Evaluation of real RNA-Seq data To demonstrate *MMR*-usage on a real RNA-Seq dataset we used a human cell line sample that has been made available in context of the RGASP project (Engström *et al.*, 2013; Steijger *et al.*, 2013). The sample can be downloaded from the Sanger ftp server [1] as file `H_sapiens-HepG2.tar` and contains 89.5×10^6 read pairs of 2×75nt length. We aligned the reads using TopHat2 and used MMR for post-processing. To evaluate performance we conducted the following two experiments. First, we used a small set of available transcript quantifications generated for this dataset using the NanoString nCounter amplification-free detection system (Kulkarni, 2011). Then we correlated the quantifications for the same transcripts estimated using *Cufflinks* with the NanoString counts for the unfiltered data, the dataset with all secondary alignments removed (keeping only the best) and the *MMR* processed alignment. The Spearman correlation coefficient decreased from 0.8323 for the unfiltered data to 0.8127 when only keeping the best alignments and remained unchanged at 0.8322 for the *MMR* processed data. Although *MMR* did not change quantification results of the 96 transcripts, more representative primary alignments were chosen. Thus, when using *MMR* to choose primary alignments instead of using the best-hit strategy, quantification results seem less affected. Note that the full BAM compressed alignment set has a total disk size of 28GB whereas the *MMR* processed version only takes 8.4GB in disk size.

For the second experiment, we aligned the same RNA-Seq data using STAR (Dobin *et al.*, 2013) and used *Cufflinks* to generate transcript quantifications. We then computed gene expression values for each gene as the sum over the predicted isoform expression values. Finally, we correlated the gene expression values generated from the STAR alignments with the ones generated from the *TopHat2* alignments. Whereas unfiltered alignments showed a Spearman correlation coefficient of 0.8496, the correlation using the best-hit alignments increased to 0.8617 and further increased for the *MMR* processed alignments to 0.8662. Although this is not a tremendous gain, it underlines *MMR*'s capability of increasing comparability between results of different alignment tools.

[1] ftp://ngs.sanger.ac.uk/production/gencode/rgasp/RGASP2/inputdata/

REFERENCES

Behr, J., Kahles, A., Zhong, Y., Sreedharan, V. T., Drewe, P., and Rätsch, G. (2013). MITIE: Simultaneous RNA-Seq-based transcript identification and quantification in multiple samples. *Bioinformatics*, **29**(20), 2529–2538.

Bohnert, R., Behr, J., and Rätsch, G. (2009). Transcript quantification with RNA-Seq data. *BMC Bioinformatics*, **10**(Suppl 13), P5.

Dobin, A., Davis, C. a., Schlesinger, F., Drenkow, J., Zaleski, C., Jha, S., Batut, P., Chaisson, M., and Gingeras, T. R. (2013). STAR: ultrafast universal RNA-seq aligner. *Bioinformatics*, **29**(1), 15–21.

Engström, P. G., Steijger, T., Sipos, B., Grant, G. R., A Kahles, Rätsch, G., Goldman, N., Hubbard, T. J., Harrow, J., Guigó, R., and Others (2013). Systematic evaluation of spliced alignment programs for RNA-seq data. *Nature Methods*, **10**(12), 1185–1191.

Griebel, T., Zacher, B., Ribeca, P., Raineri, E., Lacroix, V., Guigó, R., and Sammeth, M. (2012). Modelling and simulating generic RNA-Seq experiments with the flux simulator. *Nucleic Acids Research*, **40**(20), 10073–10083.

Hashimoto, T., De Hoon, M. J., Grimmond, S. M., Daub, C. O., Hayashizaki, Y., and Faulkner, G. J. (2009). Probabilistic resolution of multi-mapping reads in massively parallel sequencing data using mumrescuelite. *Bioinformatics*, **25**(19), 2613–2614.

Jean, G., Kahles, A., Sreedharan, V. T., De Bona, F., and Rätsch, G. (2010). RNA-Seq read alignments with PALMapper. *Current Protocols in Bioinformatics*, **Chapter 11**(December), Unit 11.6.

Kim, D., Pertea, G., Trapnell, C., Pimentel, H., Kelley, R., and Salzberg, S. L. (2013). TopHat2: accurate alignment of transcriptomes in the presence of insertions, deletions and gene fusions. *Genome Biology*, **14**(4), R36.

Kulkarni, M. M. (2011). Digital multiplexed gene expression analysis using the nanostring ncounter system. *Current Protocols in Molecular Biology*, pages 25B–10.

Lahens, N. F., Kavakli, I. H., Zhang, R., Hayer, K., Black, M. B., Dueck, H., Pizarro, A., Kim, J., Irizarry, R., Thomas, R. S., *et al.* (2014). Ivt-seq reveals extreme bias in rna sequencing. *Genome Biology*, **15**(6), R86.

Lamesch, P., Berardini, T. Z., Li, D., Swarbreck, D., Wilks, C., Sasidharan, R., Muller, R., Dreher, K., Alexander, D. L., Garcia-Hernandez, M., Karthikeyan, A. S., Lee, C. H., Nelson, W. D., Ploetz, L., Singh, S., Wensel, A., and Huala, E. (2012). The Arabidopsis Information Resource (TAIR): improved gene annotation and new tools. *Nucleic Acids Research*, **40**(Database issue), D1202–1210.

Li, B., Ruotti, V., Stewart, R. M., Thomson, J. a., and Dewey, C. N. (2010). RNA-Seq gene expression estimation with read mapping uncertainty. *Bioinformatics*, **26**(4), 493–500.

Mortazavi, A., Williams, B., and McCue, K. (2008). Mapping and quantifying mammalian transcriptomes by RNA-Seq. *Nature Methods*, **5**(7), 621–628.

Robinson, J. T., Thorvaldsdóttir, H., Winckler, W., Guttman, M., Lander, E. S., Getz, G., and Mesirov, J. P. (2011). Integrative genomics viewer. *Nature Biotechnology*, **29**(1), 24–26.

Steijger, T., Abril, J. F., Engström, P. G., Kokocinski, F., Akerman, M., Alioto, T., Ambrosini, G., Antonarakis, S. E., Behr, J., and Bertone, P. (2013). Assessment of transcript reconstruction methods for RNA-seq. *Nature Methods*, **10**(12), 1177–1184.

Trapnell, C., Williams, B. A., Pertea, G., Mortazavi, A., Kwan, G., van Baren, M. J., Salzberg, S. L., Wold, B. J., and Pachter, L. (2010). Transcript assembly and quantification by RNA-Seq reveals unannotated transcripts and isoform switching during cell differentiation. *Nature Biotechnology*, **28**(5), 511–515.

Wang, J., Huda, A., Lunyak, V. V., and Jordan, I. K. (2010). A gibbs sampling strategy applied to the mapping of ambiguous short-sequence tags. *Bioinformatics*, **26**(20), 2501–2508.

Zhang, Z., Huang, S., Wang, J., Zhang, X., de Villena, F. P. M., McMillan, L., and Wang, W. (2013). Genescissors: a comprehensive approach to detecting and correcting spurious transcriptome inference owing to rna-seq reads misalignment. *Bioinformatics*, **29**(13), i291–i299.

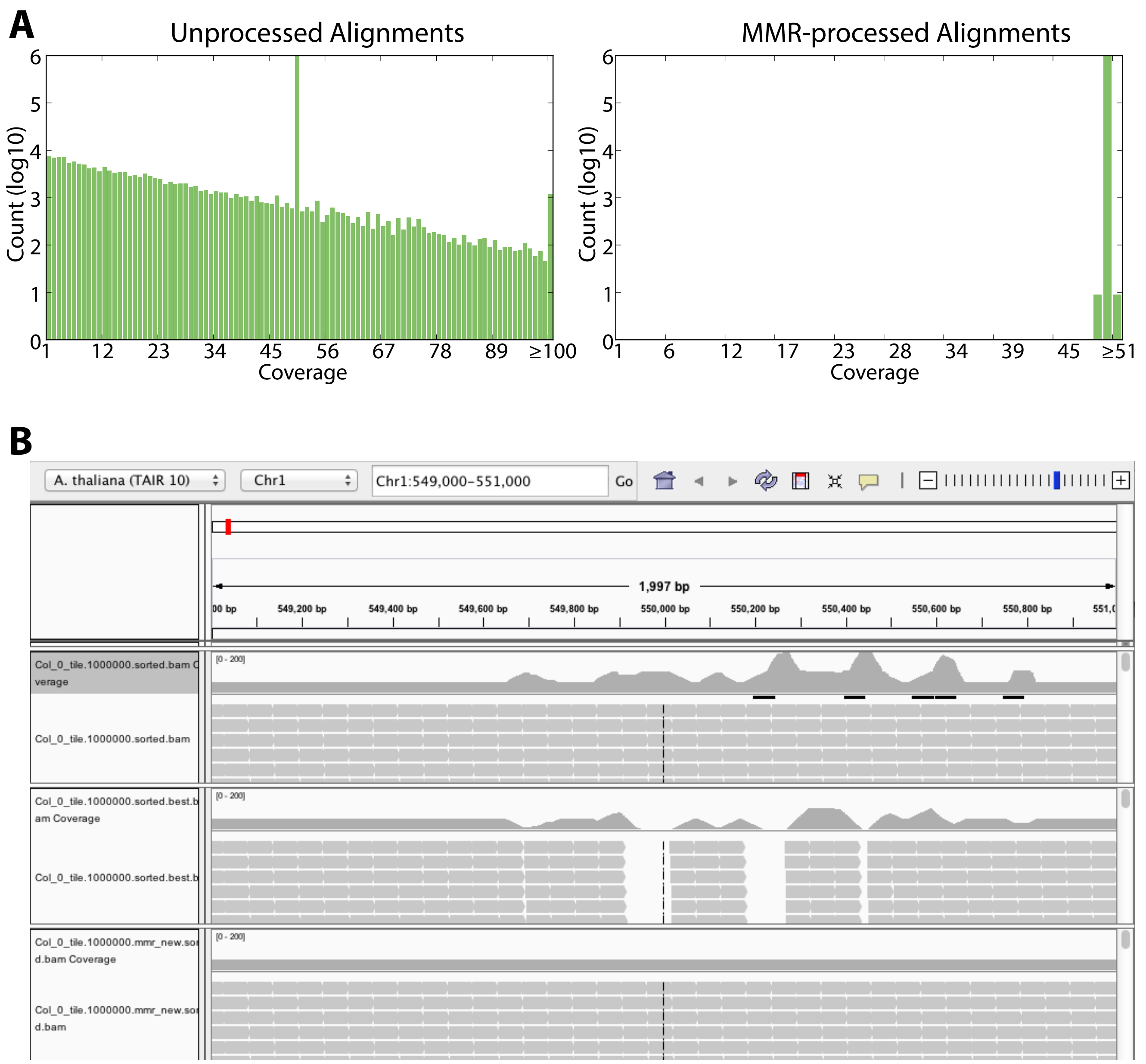

Fig. S-2: **Effect of** ***MMR*** **Smoothing —** ***A*** *MMR* effect on artificial *A. thaliana* genome tiling data. Distribution of unfiltered coverage values (left) and *MMR*-filtered values (right). ***B*** *IGV* (Robinson *et al.*, 2011) snapshot of the unfiltered (top), best-hit (middle) and *MMR*-processed (bottom) alignments. Ambiguous mappings causing unequal distributions could be fully resolved. Missing coverage caused by retaining only the best alignment cannot be observed in the *MMR* processed alignments.

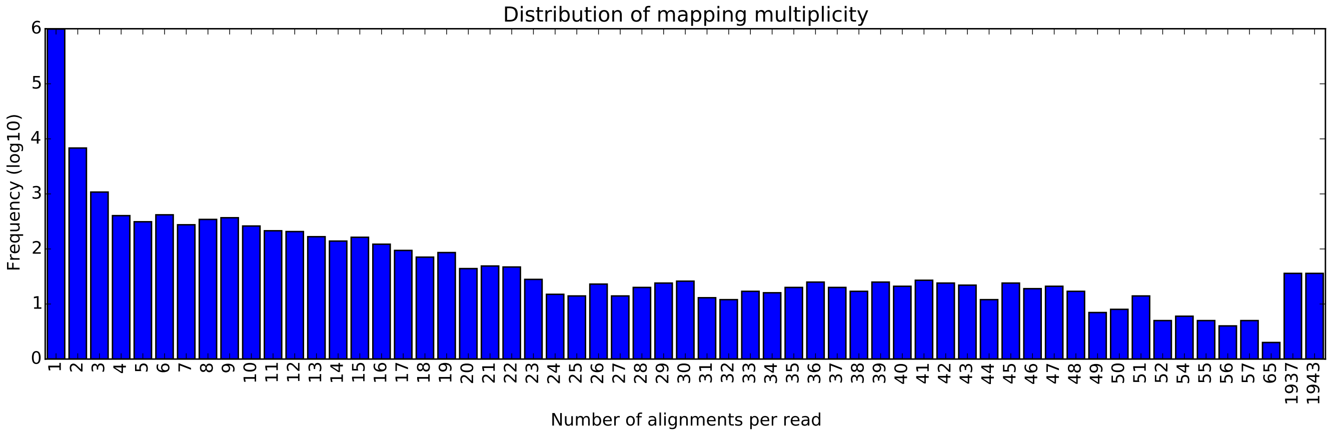

Fig. S-3: **Distribution of Alignment Multiplicity —** The above histogram shows the distribution of the number of alignment locations per read on a $\log_{10}$ scale. Most reads have exactly one alignment (most left) but a considerable number has 10 or more possible alignments. Counts are based on the artificial genome tiling data from *A. thaliana*.

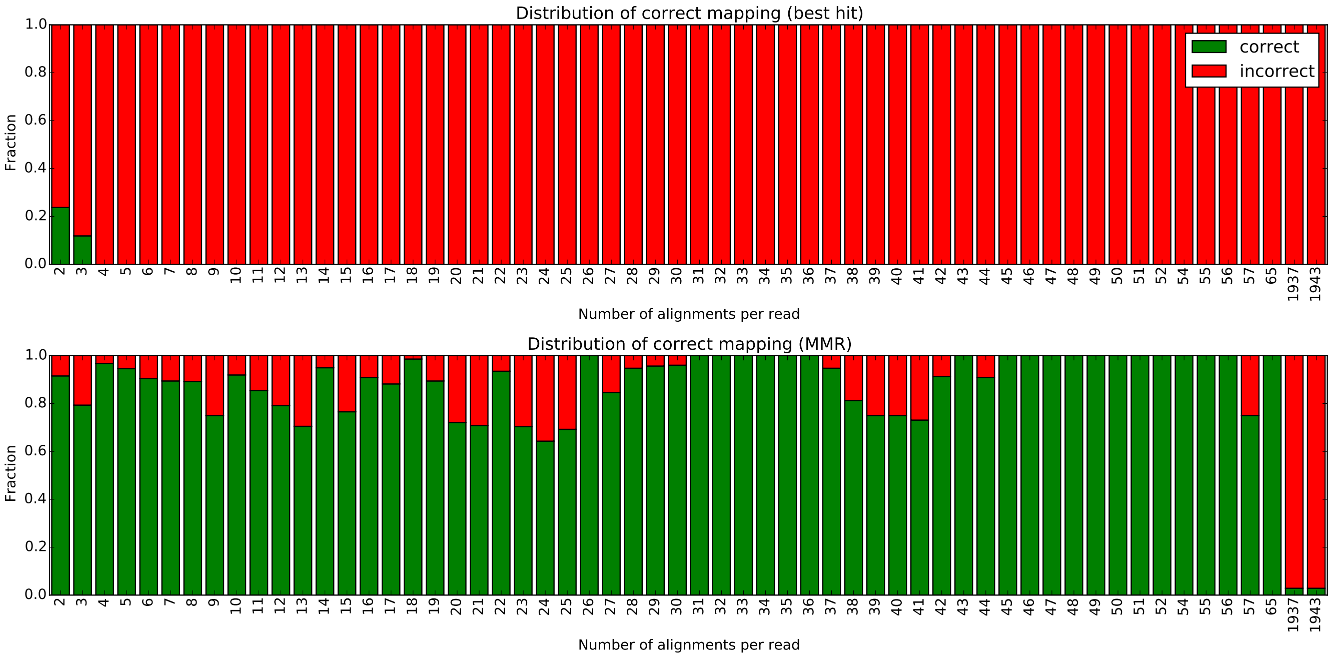

Fig. S-4: **Distribution of Correct Alignments —** For the simulated genome tiling data of *A. thaliana* the exact mapping location of each read is known. The histogram shows the fraction of reads that have been mapped to the correct location, stratified by number of possible alignment locations per read. The upper distribution is based on taking the alignment with the highest score, the lower distribution is based on *MMR*. Red indicates incorrect mapping and green indicates correct mapping. Each bar represents an alignment multiplicity stratum.

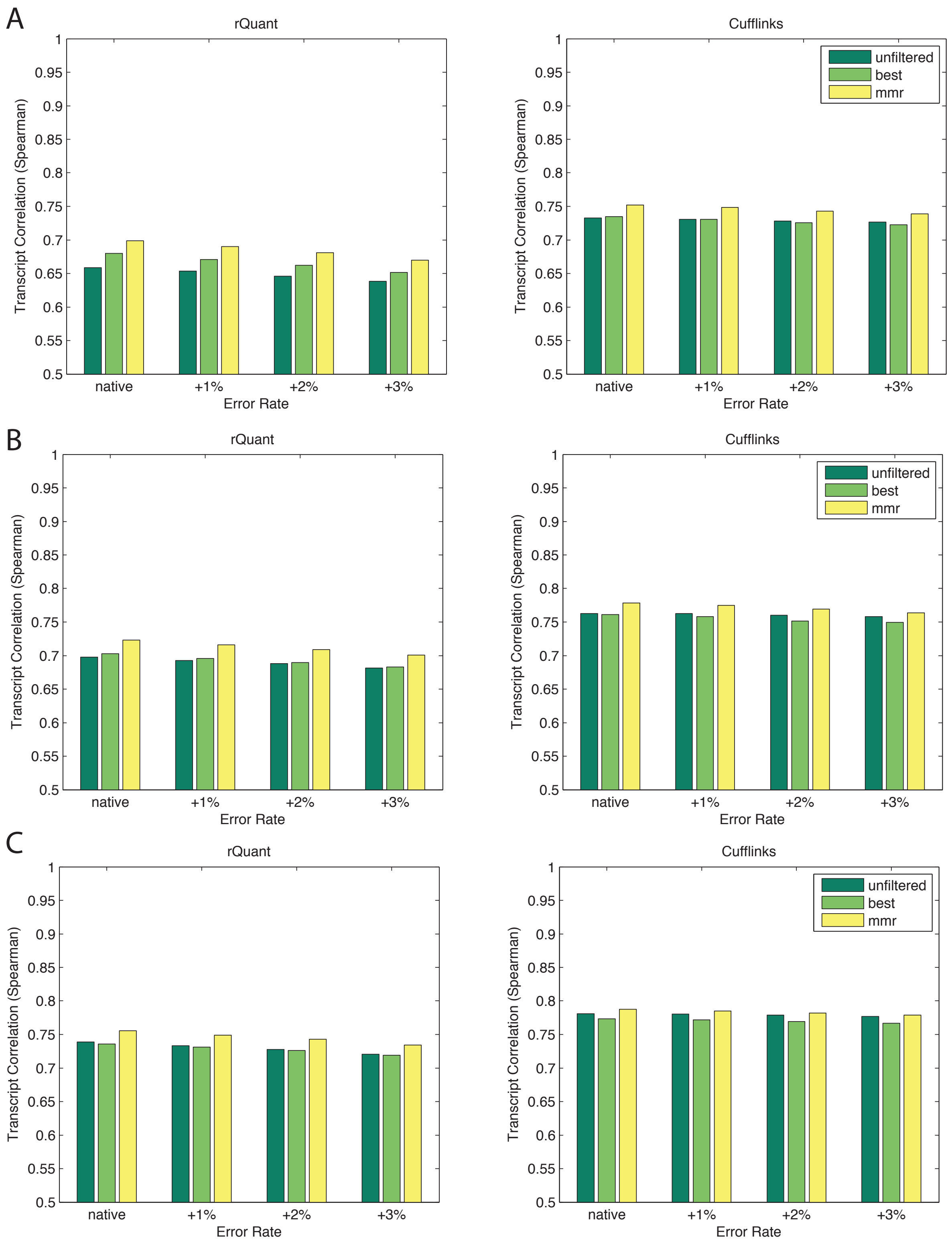

Fig. S-5: **Quantification Results for *PALMapper* Alignments —** Accuracy of predicted transcript quantifications by *rQuant* (left) and *cufflinks* (right) measured as rank correlation coefficient (Spearman) for reads of length 51nt (***A***), 76nt (***B***) and 101nt (***C***).

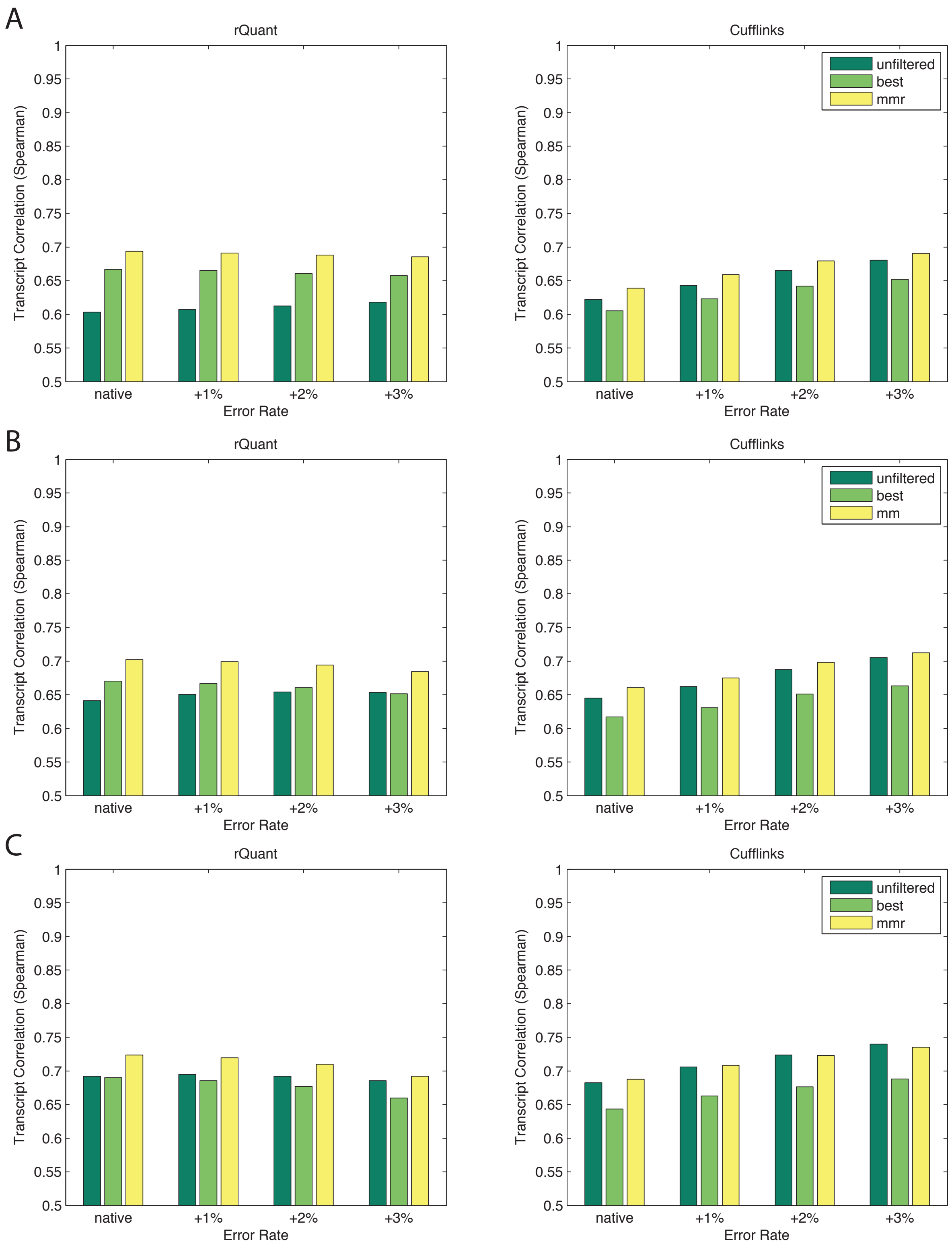

Fig. S-6: **Quantification Results for *TopHat2* Alignments —** Accuracy of predicted transcript quantifications by *rQuant* (left) and *cufflinks* (right) measured as rank correlation coefficient (Spearman) for reads of length 51nt (***A***), 76nt (***B***) and 101nt (***C***).

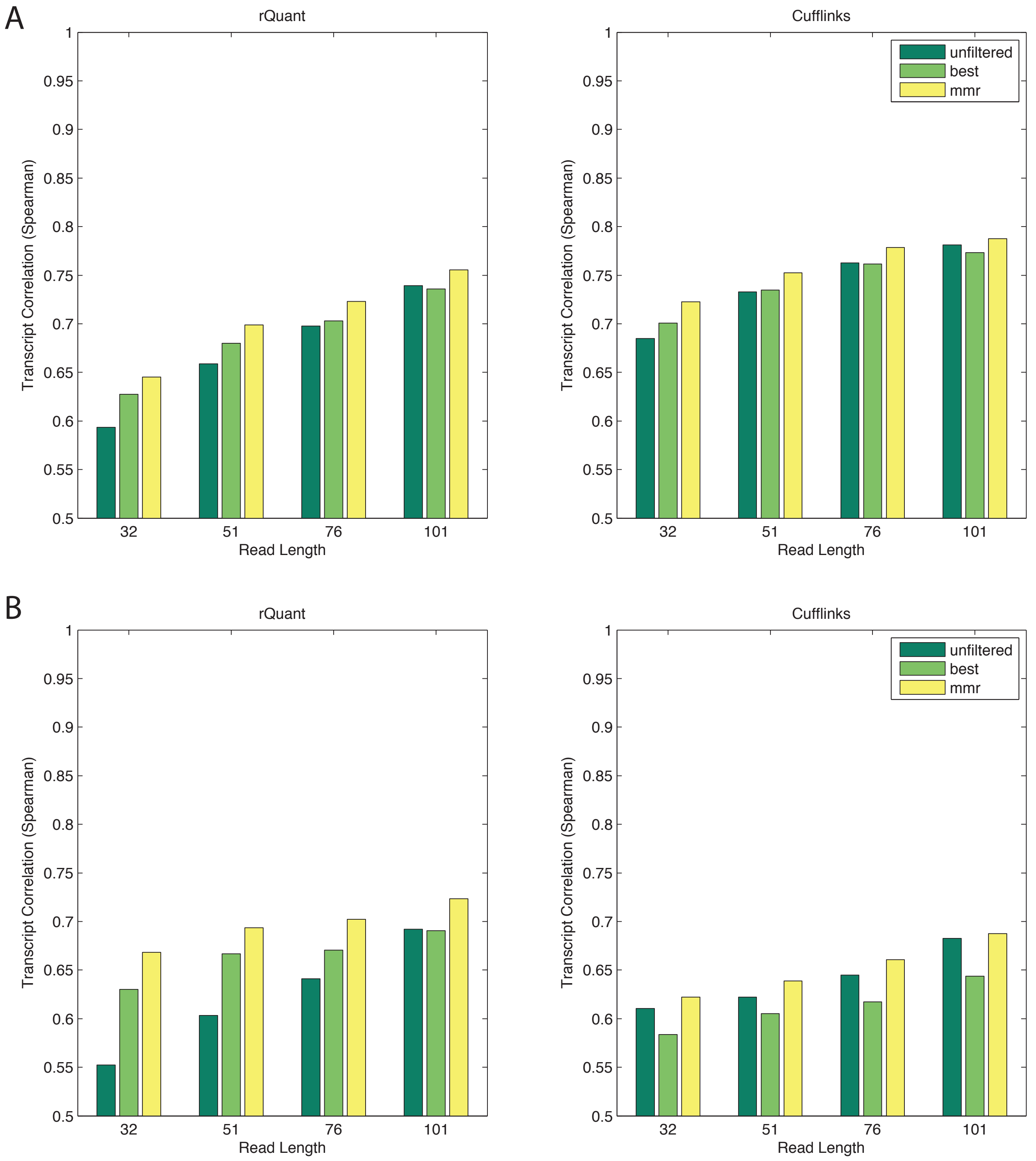

Fig. S-7: **Quantification Results dependent on Read Length —** As before, accuracy of predicted transcript quantifications by *rQuant* (left) and *cufflinks* (right) measured as rank correlation coefficient (Spearman) shown for different read lengths based on alignments with *PALMapper* (***A***) and *TopHat2* (***B***).

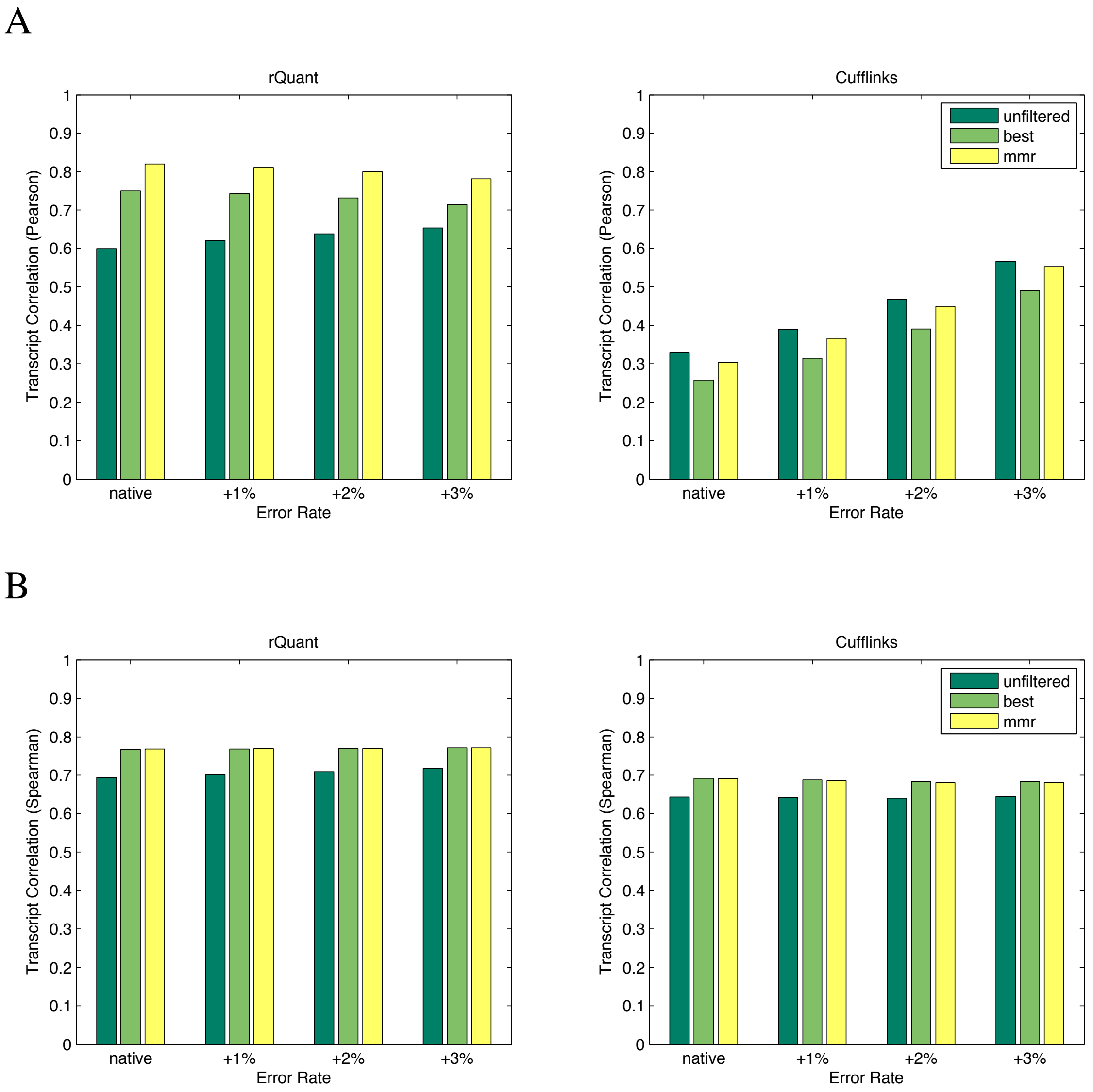

Fig. S-8: **Quantification Results on Full Annotation Simulation —** Acurracy of predicted transcript quantifications by *rQuant* (left) and *cufflinks* (right) measured as Pearson correlation (***A***) and Spearman correlation (***B***) based on alignments with *TopHat2* for 4 different error rates.

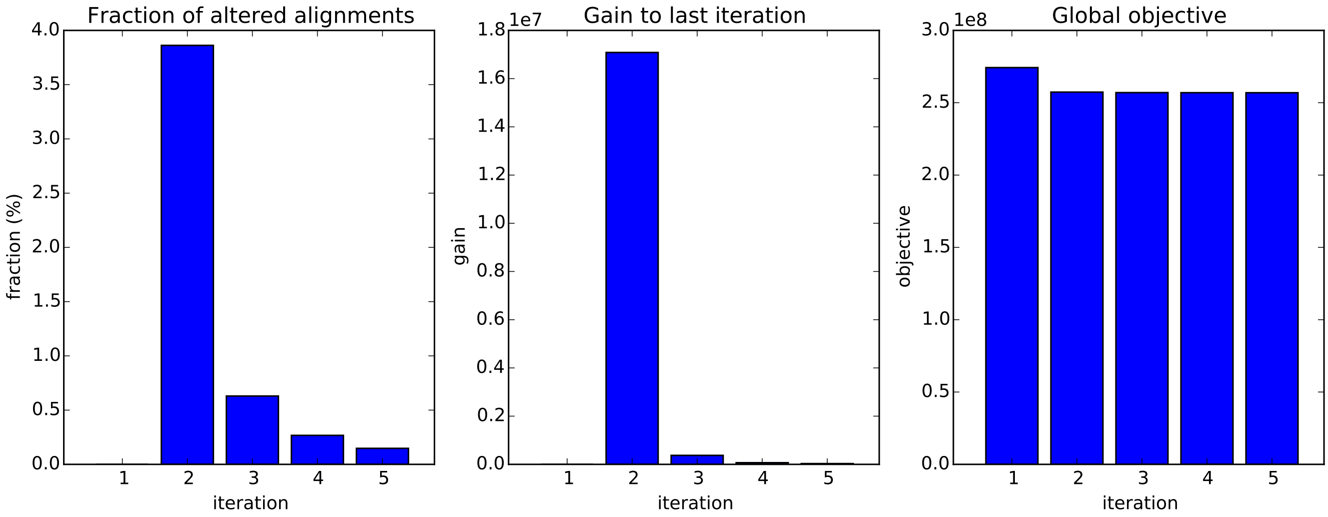

Fig. S-9: **Convergence Measures for** *MMR* **—** Three different measures of convergence for the same *MMR* run over 5 iterations. The first iteration was only used to correctly compute the global objective and no alignment was altered during this initial round. The leftmost panel shows the fraction of reads where the assignment of the alignment chosen was changed. The middle panel shows the gain (the amount the global objective is reduced) per iteration. The rightmost panel shows the global objective. Note, that only a very small fraction of read alignments is changed after the second iteration. No alignments are changed in the first iteration as this is only used to built up the coverage map for exact computation of the global objective.

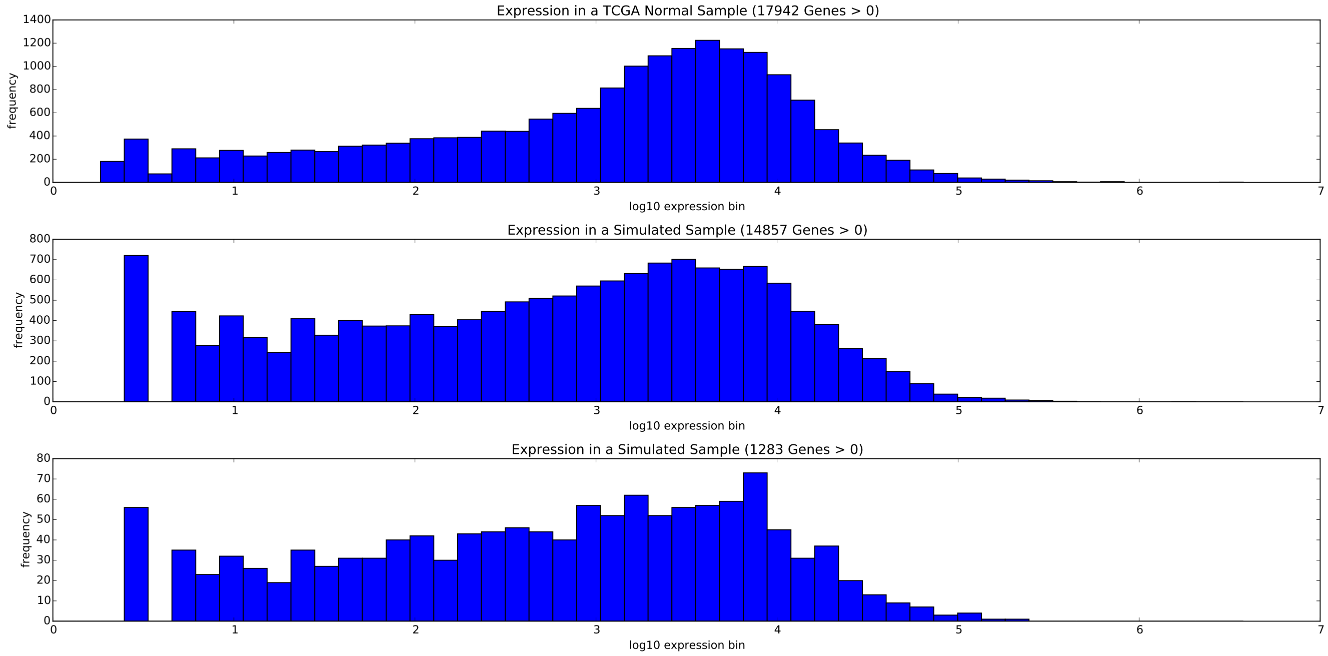

Fig. S-10: **Expression Distribution for Simulated Data —** Histograms show expression distribution for simulated and real data as $\log_{10}$ expression per gene. Genes are binned into 50 groups. The top panel shows the expression counts for a TCGA normal sample (TCGA-22-4609-11A-01R-2125-07) on 17,942 genes that had an expression count greater than 0. The middle panel shows the expression distribution created by sampling $80{\times}10^6$ reads on the Gencode annotation using *FluxSimulator* (14,857 genes with expression > 0). The lower panel shows the expression distribution for a *FluxSimulator* run, generating $7{\times}10^6$ reads from a random subset of 5,000 genes taken from the Gencode annotation (1,283 genes with expression > 0).
